# The Exosomes Derived From Bone Marrow Mesenchymal Stem Cells Alleviate Inflammatory Injury in Heart Failure Disease by Enhancing the Expression of KLF4

**DOI:** 10.1002/iid3.70161

**Published:** 2025-03-05

**Authors:** Yutong Han, Yang Bi, Donghai Zhang, Yubao Liu

**Affiliations:** ^1^ Intensive Care Unit The Second Affiliated Hospital of Qiqihar Medical University Qiqihar China

**Keywords:** exosomes, heart failure, inflammation, KLF4, mesenchymal stem cell

## Abstract

**Objective:**

The aim of this study is to investigate the impact and mechanism of action of exosomes derived from bone marrow mesenchymal stem cells (BMSCs) in the treatment of heart failure (HF).

**Methods:**

The analysis of gene sequencing data set was employed to identify potential therapeutic target proteins for HF. Subsequently, H9C2 cells and Sprague‐Dawley (SD) rats were utilized as experimental models to simulate doxorubicin hydrochloride (Doxorubicin, Dox)‐induced myocardial injury. This approach was employed to investigate the expression changes of inflammatory factors, including TNF‐α, IL‐6, IL‐1β, sST2, and Gal‐3, as well as the alterations in their expression following exosome treatment. Meanwhile, the mechanism of exosomes in relieving HF and inhibiting inflammation were investigated using constructed KLF 4 knockout cell lines and SD rats.

**Results:**

BMSC‐derived exosomes were capable of enhancing the expression level of KLF4 in cardiomyocytes, decreasing the expression levels of myocardial damage markers BNP and hs‐TnI, as well as inflammatory factors TNF‐α, IL‐6, IL‐1β, sST2, and Gal‐3, thereby alleviating HF injury. In vitro and in vivo experiments have shown that exosomal treatment decreases the expression of BNP and hs‐TnI, which are indicators of myocardial injury, along with the release of inflammatory cytokines in cardiomyocytes. Concurrently, the expression of KLF4 was downregulated, leading to a significant reduction in this physiological modulation.

**Conclusion:**

BMSC‐derived exosomes exhibit superior therapeutic potential for HF by enhancing the expression of KLF4 and mitigating inflammation in myocardial tissue.

## 1 Introduction

The syndrome of heart failure (HF) arises from impaired ventricular systole or diastole, resulting in inadequate cardiac output due to structural and functional abnormalities of the heart [1, 2]. The incidence of HF is currently on the rise, with an annual increase observed. Moreover, the 5‐year mortality rate among patients exceeds 50%, making it a pivotal concern within the realm of clinical medicine [3, 4]. The administration of various therapeutic agents has resulted in a partial alleviation of HF symptoms; however, it does not modify the progression of HF or enhance patients' quality of life [5]. Therefore, effective treatments and novel therapeutic targets for HF still need to be discovered and researched.

HF leads to a substantial elevation in inflammatory markers in patients, significantly diminishing their quality of life [6, 7]. The expression of various pro‐inflammatory factors is absent or limited in normal cardiac tissue, whereas their upregulation can be observed in damaged myocardium [8, 9]. In recent years, numerous studies have demonstrated that pro‐inflammatory cytokines play a pivotal role in the pathogenesis and progression of HF [10]. The excessive activation and subsequent cascade response of pro‐inflammatory cytokines can inflict severe physiological damage on patients. pro‐inflammatory factors promote left ventricular remodeling in the heart, resulting in reduced myocardial contractility, myocardial hypertrophy, and pathological changes such as myocardial fibrosis. Furthermore, pro‐inflammatory cytokines elevate the risk of atherosclerosis and trigger oxidative stress in endothelial cells. Concurrently, the elevated expression of these cytokines can also lead to metabolic dysregulation in skeletal muscle, thereby promoting apoptosis and contributing to the development of cachexia [11, 12].

Mesenchymal stem cells (MSCs) possess a remarkable capacity for self‐renewal and multi‐lineage differentiation, rendering them valuable as cellular substrates in tissue engineering applications [13, 14, 15, 16, 17, 18, 19]. Although bone marrow‐derived MSC (BMSCs) therapy has demonstrated certain efficacy in the treatment of heart failure, the precise underlying mechanism remains elusive [20, 21].

Exosomes are vesicular structures secreted by living cells, typically measuring between 50 and 150 nm in diameter. These vesicles are rich in various bioactive components, including DNA, RNA, proteins, and lipids [22, 23].

MSC and its secretions exhibit substantial cardioprotective effects. In the treatment of myocardial infarction/ischemia, fibrosis, hypertrophy, dilated cardiomyopathy and atherosclerosis and other cardiac diseases, MSC have shown excellent immunomodulatory functions. On the one hand, its own ability to directly differentiate into cardiomyocytes and epidermal cells to improve the body's efficiency of damage repair. On the other hand, the exosomes secreted by MSCs can significantly enhance vascular density, promote cardiac remodeling, and improve ejection fraction via paracrine mechanisms. Additionally, these exosomes can reduce apoptosis, minimize wound size, lower end‐diastolic and end‐systolic volumes, and prevent fibrosis and oxidative stress. Several pro‐neovascular factors are present in BMSC secretions, including fibroblast growth factor 2 (FGF‐2), vascular endothelial growth factor (VEGF), monocyte chemoattractant protein‐1 (MCP‐1), angiopoietin‐1 (Ang‐1), and placental growth factor (PLGF), which can effectively improve the body's repair ability in the face of cardiac injury. Furthermore, exosomes secreted by MSCs also contain long noncoding RNAs (lncRNAs), such as miR‐210. These lncRNAs do not encode proteins but are capable of participating in the regulation of gene expression and promoting tissue repair [24]. However, the specific mechanisms and physiological functions of exosomes in HF treatment still need to be further investigated.

Krüppel‐like factor 4 (KLF4) is a eukaryotic transcription factor that has been shown to be involved in the occurrence and development of several physiological activities and pathological changes [25, 26, 27]. It has been shown that KLF4 can play a protective role in acute lung injury, and upregulating KLF4 enhances the pathological state and inflammatory response in LPS‐induced acute lung injury in mice. The expression of KLF4 also enhances the protective effect on meniscal cells and synoviocytes, while promoting chondrogenic differentiation of MSCs. The KLF4‐dependent regulation of smooth muscle cell phenotype plays a pivotal role in the pathogenesis of atherosclerotic plaques. The inhibition of miR‐25 in a mouse model of HF resulted in an increased expression level of KLF4, thereby improving cardiac function through the attenuation of fibrosis. Although KLF4 plays a multitude of physiological roles, its involvement in the development of HF has not been thoroughly investigated. Despite the extensive verification of biological activities associated with BMSC‐derived exosomes, their specific mechanism of action in treating HF and alleviating inflammatory response remains unclear. The objective of this study is to investigate the therapeutic effects of exosomes derived from BMSCs in HF and elucidate their mechanism of action, aiming to provide a theoretical basis and directional guidance for HF treatment and inflammation control.

The present study demonstrates that exosomes derived from BMSCs possess physiological functions in attenuating cardiomyocyte injury and reducing inflammatory response levels, suggesting their potential as an effective therapeutic modality for HF. However, further comprehensive investigations are required to elucidate the specific regulatory mechanisms involved. Additionally, KLF4 may represent a promising target for HF therapy.

## 2 Methods

### 2.1 Construction and Grouping of Cell Models

To verify the in vitro damage caused by Dox to cardiomyocytes and to evaluate the reparative potential of exosomes, rat H9C2 cells were utilized as experimental materials. The cells were cultured in a 37°C constant temperature incubator with 5% CO_2_ using DMEM medium containing 10% FBS, and the in vitro myocardial injury cell model was established by adding Dox solution to the H9C2 cells in the constant temperature incubator.

The in vitro experiments included four groups: control group, injury group, treatment group, and KLF4‐KO treatment group. In the control group, wild‐type H9C2 cells were used in the control group, and no Dox was added during the culture process. In the injury group, wild‐type H9C2 cells were utilized, and the group was exposed to 5 μmol/L Dox for 24 h, modeling was deemed successful when cell viability approached 50%, and there was no significant alteration in cell morphology. Following successful modeling, the injury group was administered with PBS and further cultured for an additional 12 h to serve as a control. PBS was administered to the injury group as a control for 12 h. The treatment group used wild‐type H9C2 cells, which were treated with 5 μmol/L Dox for 24 h, after successful modeling, an equal volume of exosomes was added for treatment, followed by continued incubation for 12 h. The KLF4‐KO treatment group used KLF4‐KO H9C2 cells, which were treated with 5 μmol/L Dox for 24 h, after successful modeling, an equal volume of exosomes was added for treatment, followed by continued incubation for 12 h.

### 2.2 Construction of H9C2 Cell Line With KLF4‐KO

To construct the KLF4‐KO H9C2 cells, KLF4 was knockdown by lentiviral packaging system in H9C2 cells. For the experiment, an appropriate number of wild‐type H9C2 cells were seeded in 6‐well plates and cultured in a 37°C constant temperature incubator with 5% CO_2_ using DMEM medium supplemented with 10% FBS. Lentiviral infection was conducted when the cell density reached 30%. Following the removal of the complete medium, a fresh, double‐antibody‐free medium along with the lentiviral solution was added. After the cells were infected for 12 h, they were washed and replaced with complete medium. Puromycin was then added 48 h postinfection and maintained for an additional 48 h. The cell lines obtained from the screening were examined for the expression level of KLF4, and were passaged and frozen.

### 2.3 Construction and Grouping of Animal Models

To analyze the changes of BNP and hs‐TnI, and TNF‐α, IL‐6, IL‐1b, sST2, and Gal‐3 in animals after myocardial damage, 40 SD rats were chosen at 6‐8 weeks of age, and after 2 weeks of routine feeding, they were divided into four groups of 10 rats each. During feeding and modeling, the ambient temperature was maintained at 24 ± 2°C and the relative humidity at 50% ± 10%. A 12‐h light‐dark cycle was implemented to ensure optimal conditions for the rats' free movement, feeding, and drinking.

The control group used wild‐type SD rats, which were injected three times intraperitoneally with 0.9% NaCl solution according to 15 mg/kg at 2‐week intervals for 6 weeks, and then injected once a week later in the tail vein with 100 µL of 0.9% NaCl solution, and then continued to be fed for another 3 weeks. The injury group used wild‐type SD rats, In the injured group, wild‐type SD rats were used, following three intraperitoneal injections of 0.5 mg/mL Dox solution at 15 mg/kg, injected at 2‐week intervals for 6 weeks, and one 100 µLPBS injection in the tail vein 1 week after modeling, and then continued feeding for another 3 weeks. The treatment group used wild‐type SD rats with three injections of 0.5 mg/mL Dox solution for 6 weeks, 100 µL exosome suspension for 3 weeks. In the KLF 4‐KO treatment group, SD rats with knockdown KLF 4 were injected with 0.5 mg/mL Dox solution at 15 mg/kg at 2 weeks, 100 µL exosome suspension in the tail vein after modeling, and continued feeding for another 3 weeks.

The cardiac function of rats was examined using small animal cardiac ultrasound, and a left ventricular ejection fraction (LVEF) < 50% was used as a criterion for successful modeling.

### 2.4 Construction of KLF4‐KO SD Rats

The SD KLF4‐KO rats were purchased from Sayer Biologicals, and the construction was relied on the principle of CRISPR technology. The main process included the construction of gRNA vector, the preparation of mRNA, microinjection, the construction of the F0 generation mice, and the construction of the F1 generation mice.

### 2.5 Collection of Clinical Serum Samples

Clinical serum samples: serum samples from 50 patients with clinical heart failure, and serum samples from 50 healthy volunteers. This study was approved by the Ethics Committee of the Second Affiliated Hospital of Qiqihar Medical University (202402003‐01), and all the patients had signed the informed consent form.

### 2.6 Reagents

Rat myocardial H9C2 cell (CRL‐1446), human BMSCs (PCS‐500‐012) were purchased from ATCC. DMEM medium (C11995500BT), fetal bovine serum (FBS, 10099‐141) were purchase from Gibco, USA. Lentiviral vector plasmid (GV341) was purchased from Shanghai Jikai, Trizol reagent (R0016), β‐actin (A5092) antibody were purchased from Beyotime (Shanghai, China). Annexin V‐FITC Apoptosis Detection Kit (APOAF) was purchased from Sigma, USA. Reactive oxygen species (ROS) ELISA kit (LZ‐E030167) was purchased from Shanghai Lianzu. Brain natriuretic peptide (BNP) ELISA kit (XG‐E988530) was purchased from Shanghai SIG Biotechnology Co. Ultrasensitive Troponin I (hs‐TnI) ELISA kit (20222400122) was purchased from Shanghai Kehua Bioengineering Co. Interleukin 6 (IL‐6) ELISA kit (ab178013), Interleukin 1β (IL‐1b) ELISA kit (ab217608), CD9 antibody (13174), CD63 antibody (52090), TSG101 antibody (72312), Alix antibody (92880), HSP70 antibody (4872), Calnexin antibody (2433) were purchased from Abbott Antibody (Shanghai) Trading Co. H3 antibody (4499), GM130 antibody (12480), Goat anti‐rabbit IgG H&L (HRP) (ab205718), Goat anti‐mouse IgG H&L (HRP) (ab205719) was purchased from Abbott Antibody (Shanghai) Trading Co. KLF4‐KO rats were purchased from Sayer Biologicals, and RNA rapid extraction solution (AM9775) was purchased from Invitrogen.

### 2.7 GEO Database Analysis

The HF data set GSE253984 was downloaded from the GEO database (http://www.ncbi.nlm.nih.gov/geo), and *p* < 0.001 was taken to obtain the differentially expressed genes for differential expression analysis using t‐test. The screening of differentially expressed genes was based on the criteria of | log2(Fold Change) | ＞ 1, *p* < 0.001 as the criterion. Differential analysis was performed using the R software Limma package. Pearson correlation method was used for correlation analysis. *p* < 0.05 was considered statistically different.

### 2.8 ELISA

The patient serum, H9C2 cells, rat myocardial tissue or rat serum, and standards were incubated at room temperature and subjected to a gradient dilution process. All samples and standards were sequentially added to the sample wells of the ELISA plate under different dilution conditions (1*10, 1*10^2^, 1*10^3^, 1*10^4^, 1*10^5^, 1*10^6^). After incubation at 4°C for 8 h, thorough washing was performed followed by addition of HRP enzyme marker. Subsequently, after further incubation at 4°C for 2 h, another round of thorough washing was carried out and absorbance against OD 450 nm was measured using an enzyme marker. The standards were utilized to construct a standard curve, enabling the determination of the precise amount of target protein in each sample.

### 2.9 Extraction of Exosomes

In this experiment, ultracentrifugation was employed for exosome extraction from BMSC. After resuscitation and culturing of BMSCs to the third generation, when 80% cell fusion was observed under microscopic examination, complete medium without exosome serum was substituted, and the supernatant was collected after 36–48 h of culture. The first step involved the removal of dead cells through centrifugation at 4°C, 300xg. Subsequently, the cells were centrifuged at 4°C and 2000 × *g* for 10 min, followed by transfer to an ultracentrifuge tube. Further centrifugation was performed at 10,000 × *g* for 30 min to eliminate cellular debris and obtain exosomes. Finally, the exosomes were resuspended with PBS to yield the exosome suspension.

### 2.10 Identification of Exosomes

The morphological structure of exosomes was visualized using transmission electron microscopy (Thermo ScientificTM TalosTM F200X S/TEM). The exosomes were fixed using a 2.5% glutaraldehyde solution, followed by the dropwise addition of 5 µL of exosome suspension onto the copper mesh. Excess liquid was then aspirated and dried, and subsequently stained with lead citrate solution, washed, dried, and observed under a microscope.

The particle size of exosomes was analyzed using nanoparticle tracking analysis. Before measurement, the sample cell underwent cleaning with deionized water, while the equipment was calibrated using 100 nm polystyrene microspheres. Subsequently, diluted exosome suspensions were subjected to on‐board inspection following washing in PBS buffer.

### 2.11 RT‐PCR

RNA was extracted from H9C2 cells or rat myocardial tissues using RNA Rapid Extraction Solution (AM9775, Invitrogen). The RNA was subsequently reverse‐transcribed into cDNA using the BioScript All‐in‐One cDNA Synthesis SuperMix (Bimake, Houston, TX). RT‐PCR analysis was performed using FastStart Universal SYBR Green Master Mix (Roche, Mannheim, Germany) and CFX96 TM Real‐time PCR System (Bio‐Rad, Hercules, CA). The relative expression levels of RNAs were calculated using 2‐(^ΔΔ^Ct). Primer sequences were in Table 1.

**Table 1:** Primer sequences.

Gene	Forward primer, 5′–3′	Reverse primer, 5′–3′
TNF‐α	GCTCTTACTGACTGGCATGAG	CGCAGCTCTAGGAGCATGTG
IL‐6	GCCTTGCTCGGCAAGTAGAT	TCCTCCGTTTCAGCCAGTTT
IL‐1b	AGAGCGGGCTGAATGCAATG	ATATTCTGCCATGCCAGCTTCAG
sST2	CCAAGGCAAGAGATCAAACCCA	CAGGATGATTCCGCAGAGGAT
Gal‐3	CCTTCCCCTGCAACCAGTTT	CCACGCAGCCGTTCTTATCA
β‐actin	GGCTGTATTCCCCTCCATCG	CCAGTTGGTAACAATGCCATGT

### 2.12 Western‐Blot

Total proteins were extracted from H9C2 cells or rat myocardial tissues using RIPA cell lysate and collected by centrifugation. Protein concentration was determined using the BCA method. After quantifying the protein concentration, SDS‐PAGE electrophoresis was performed on each tissue or cell sample. Then the separated proteins were transferred onto a PVDF membrane. Subsequently, the PVDF membrane was blocked with 5% skimmed milk for 1 h and washed with TBS. The primary antibody against the marker protein was then incubated overnight at 4°C. Afterwards, the secondary antibody was added and incubated for 1 h. Finally, chemiluminescent imaging system was used to detect signals from the exposed PVDF membrane. The names and specifications of both primary and secondary antibodies used were consistent with those described in the experimental reagents.

### 2.13 Statistical Analysis

GraphPad Prism9 software was applied for statistical analysis of data. All data were tested for normality, and data that conformed to normal distribution were expressed as mean ± standard deviation (mean ± SD). Statistical differences between the means of two groups were compared using *t*‐test, and statistical differences between the means of multiple groups were compared using one‐way ANOVA. Correlation analysis was performed using the Pearson correlation method. *p* < 0.05 was considered statistically different.

## 3 Results

### 3.1 The Expression of KLF4 was Downregulated in Patients With HF

**Figure 1:**
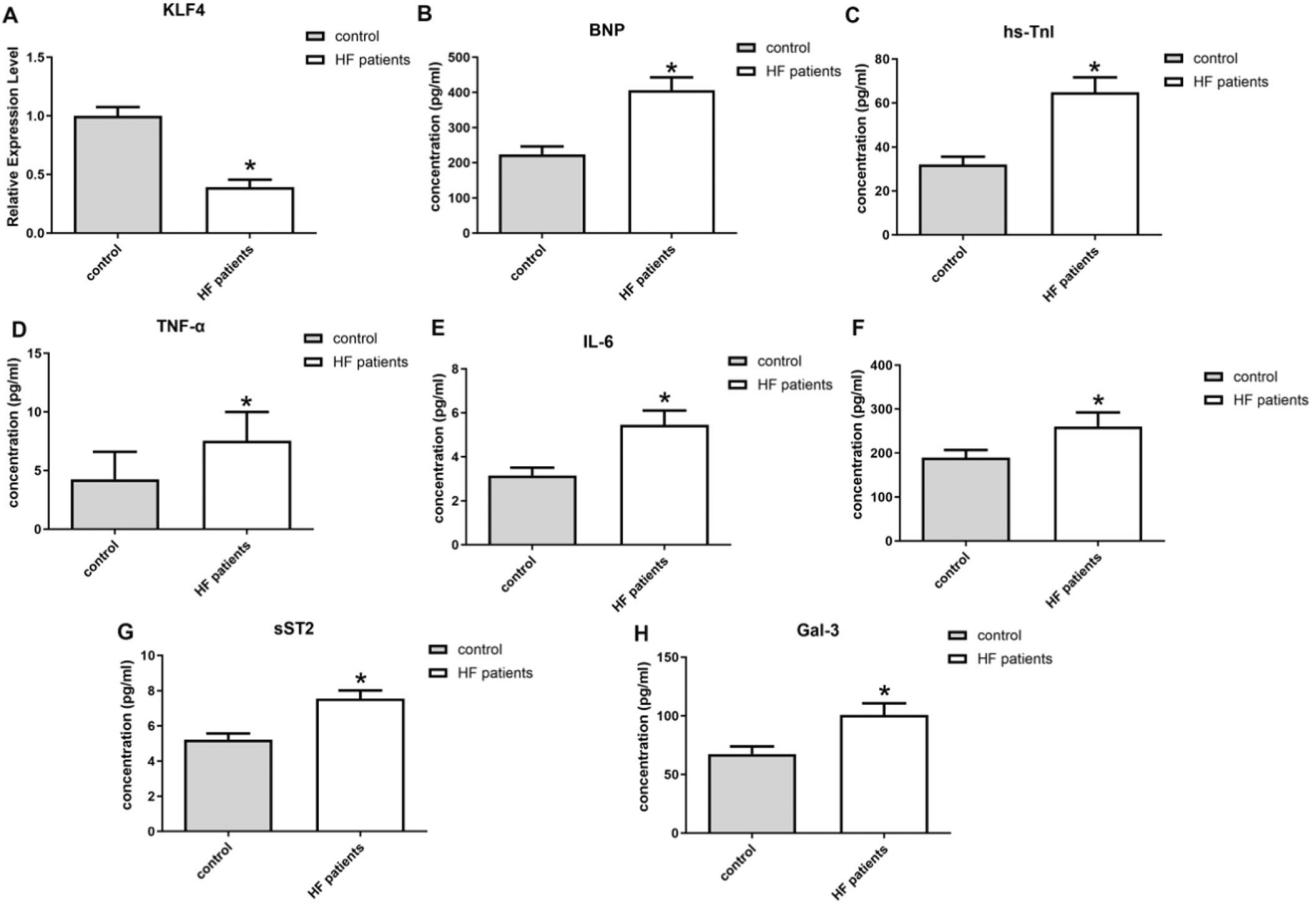
GSE253984 analysis of the HF gene sequencing data set. (A) The comparison of KLF4. (B) ELISA for BNP; (C) ELISA for hs‐TnI; (D) ELISA for TNF‐α; (E) ELISA for IL‐6; (F) ELISA for IL‐1b; (G) ELISA for sST2; (H) ELISA for Gal‐3.

The analysis of the HF gene sequencing data set GSE253984 revealed a significant downregulation in the expression of KLF4 among patients with HF compared to the normal group (Figure 1A). Serum samples from patients with clinical HF revealed that the markers of myocardial damage, BNP and hs‐TnI, showed significantly elevated expression (Figure 1B,C). Meanwhile, in the serum of HF patients, the expression of several inflammatory cytokines appeared substantially elevated, including tumor necrosis factor‐α (TNF‐α), interleukin‐6 (IL‐6), interleukin‐1b (IL‐1b), soluble suppression of tumorigenicity‐2(sST2), and Galectin‐3(Gal‐3) (Figure 1D–H). The findings indicated a strong association between the inflammatory response and disease progression in HF.

### 3.2 Extraction and Characterization of BMSC‐Derived Exosomes

**Figure 2:**
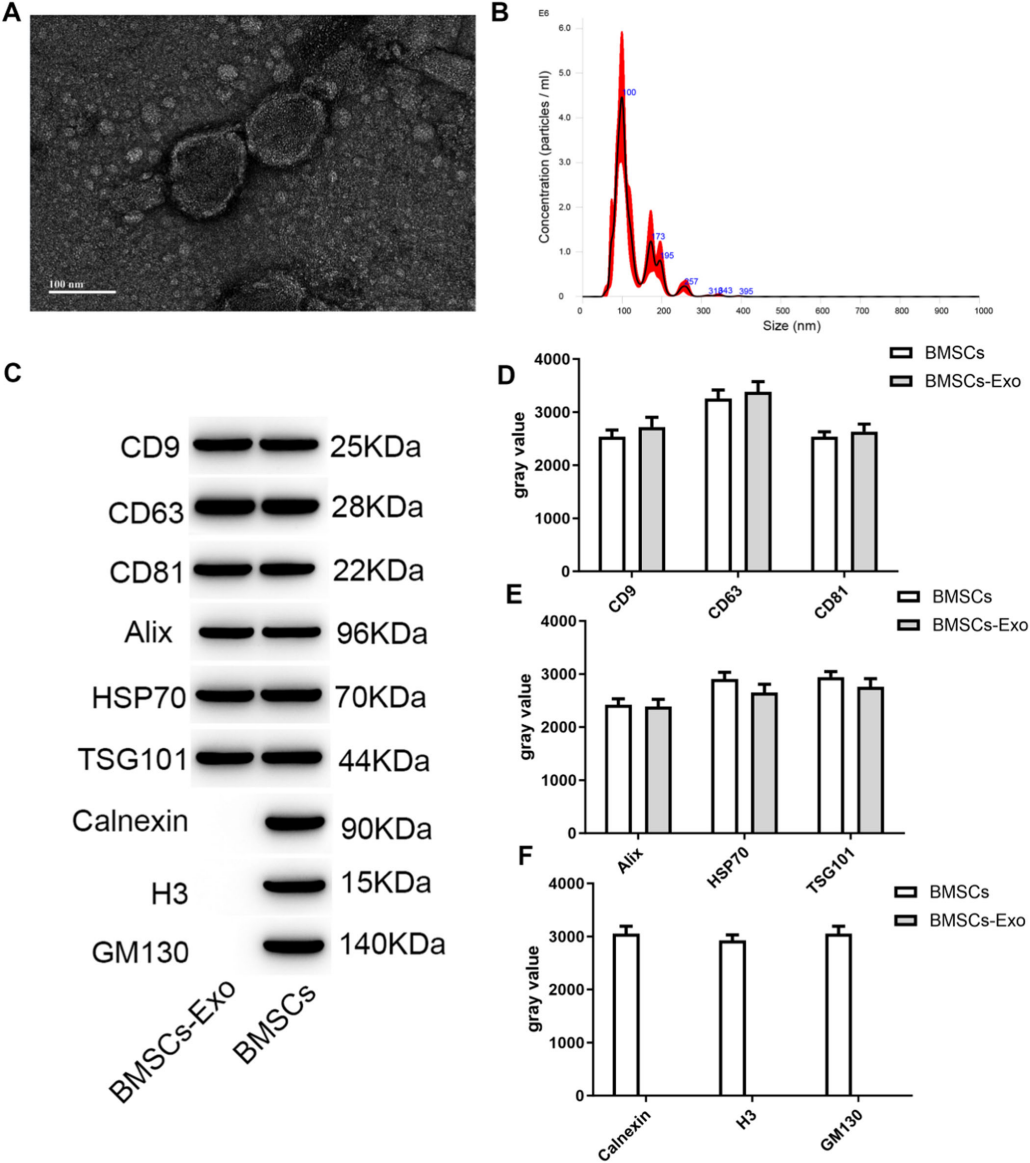
Characterization of exosomes from BMSCs. (A) Electron microscopic structure of exosomes; (B) Exosome particle size distribution; (C) Western blot for CD9, CD63, CD81, TSG101, Alix, HSP70, Calnexin, H3, GM130; (D) Western blot quantitative analysis of CD9, CD63, CD81; (E) Western blot quantitative analysis of TSG101, Alix, HSP70; (F) Western blot quantitative analysis of Calnexin, H3, GM130.

The exosomes isolated through ultracentrifugation exhibited a characteristic cup‐shaped morphology (Figure 2A), with particle sizes uniformly distributed within the range of 50‐150 nm (Figure 2B), showcasing typical structural features of exosomes. Meanwhile, exosomes exhibited the characteristic exosome proteins, including typical transmembrane proteins CD9, CD63, and CD81 (Figure C), as well as lysosomal proteins TSG101, Alix, and HSP70 (Figure 2D). Conversely, they did not demonstrate the presence of endoplasmic reticulum protein Calnexin, cytosolic protein H3, or Golgi protein GM130 (Figure 2E).

### 3.3 The BMSCs Exosome Treatment Enhanced the Expression of KLF4 and Reduces the Expression of Inflammatory Factors in H9C2 Cells

**Figure 3:**
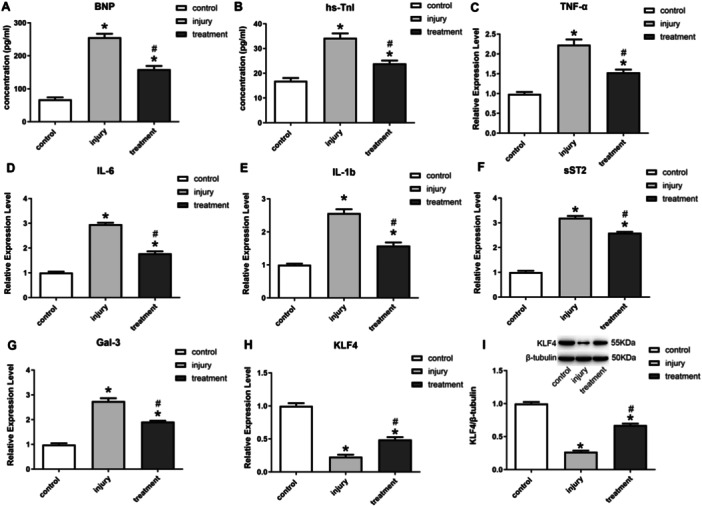
The BMSCs exosome treatment enhances the expression of KLF4 and reduces the expression of inflammatory factors in H9C2 cells. (A, B) ELISA for BNP, hs‐TnI. (C–H) qPCR for TNF‐α, IL‐6, IL‐1b, sST2, Gal‐3, KLF4; (I) Western blot for KLF4.

Compared to normal cells, Dox induced significant damage in H9C2 cells, as evidenced by the upregulation of cardiomyocyte damage markers BNP and hs‐TnI (Figure 3A,B), as well as the increased expression of inflammation‐related cytokines TNF‐α, IL‐6, IL‐1b, sST2, and Gal‐3 (Figure 3C–G). Meanwhile, the expression of KLF4 and inflammatory cytokines were examined in the cells after treatment with BMSC‐derived exosomes. The findings revealed a significant upregulation of KLF4 expression in H9C2 cells subsequent to exosome treatment (Figure 3H,I). Meanwhile, compared with the control group, exosome treatment was able to significantly down‐regulate the expression levels of BNP and hs‐TnI (Figure 3A,B), as well as the expression levels of inflammatory factors in the cells (Figure 3C–G).

### 3.4 Inhibition of KLF4 Reduced the Level of Inflammatory Relief in Exosomes

**Figure 4:**
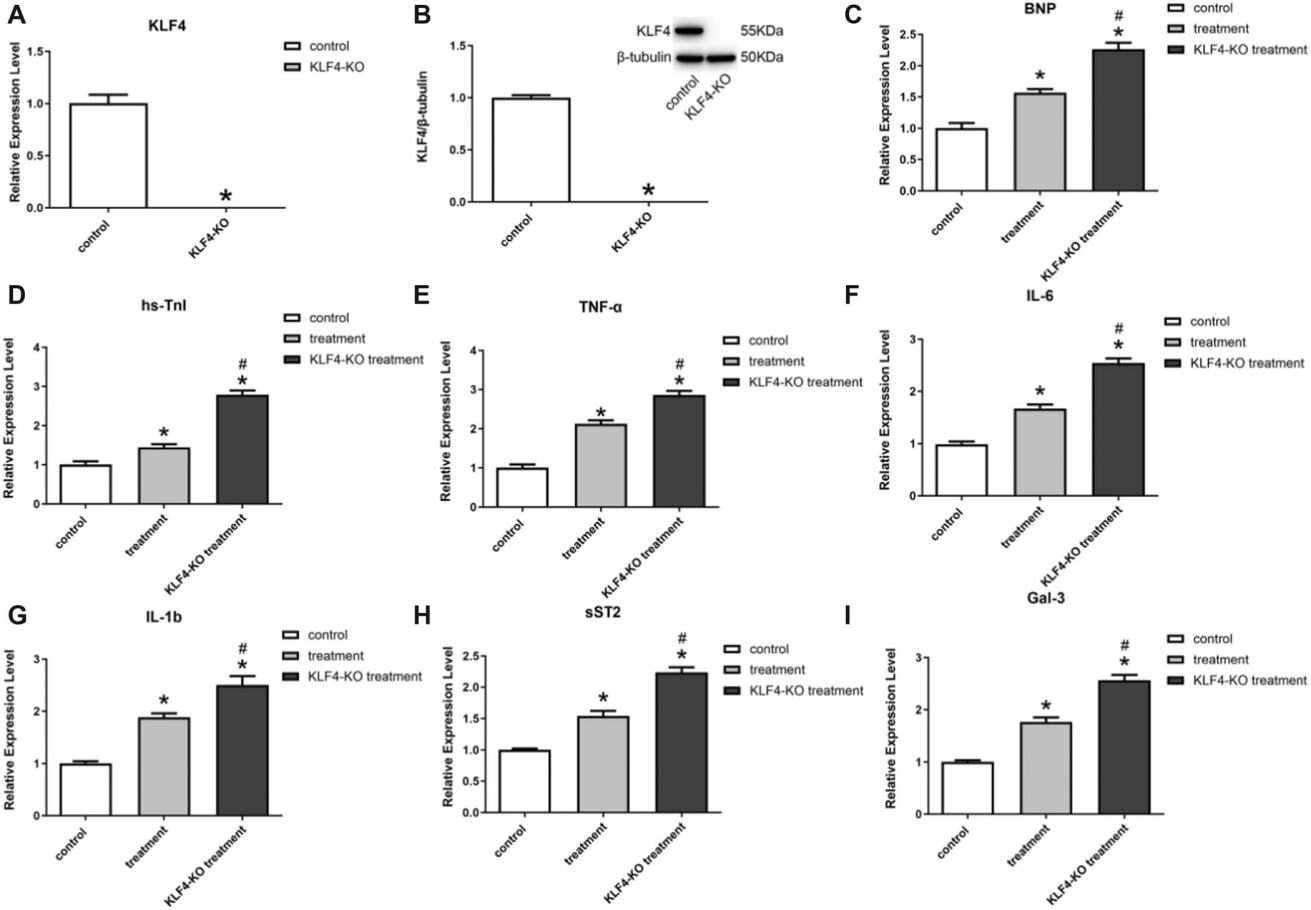
Inhibition of KLF4 reduced the level of inflammatory relief in exosomes. (A) qPCR for KLF4; (B) Western blot for KLF4; (C–I) qPCR for BNP, hs‐TnI, TNF‐α, IL‐6, IL‐1b, sST2, Gal‐3.

To investigate the relationship between the inhibitory inflammatory function of exosomes and the KLF4 factor, this study constructed a cell line with knockdown of the KLF4(KLF4‐KO), and the results showed that KLF4 was completely suppressed in H9C2 cells with KLF4‐KO (Figure 4A,B). After Dox‐induced injury, the expression of myocardial injury marker proteins BNP and hs‐TnI was found to be elevated in KLF4‐KO cells following exosome treatment, as compared to normal cells (Figure 4C,D), while the expression of TNF‐α, IL‐6, IL‐1b, sST2, and Gal‐3 were elevated (Figure 4E–I), suggesting that the inflammation inhibition of BMSC‐derived exosomes ability appeared significantly reduced, thus demonstrating that the therapeutic effect of exosomes on damaged cardiomyocytes was realized through KLF4.

### 3.5 BMSC Exosome Treatment Promoted KLF4 and Suppresses Inflammatory Responses in HF Rats

**Figure 5:**
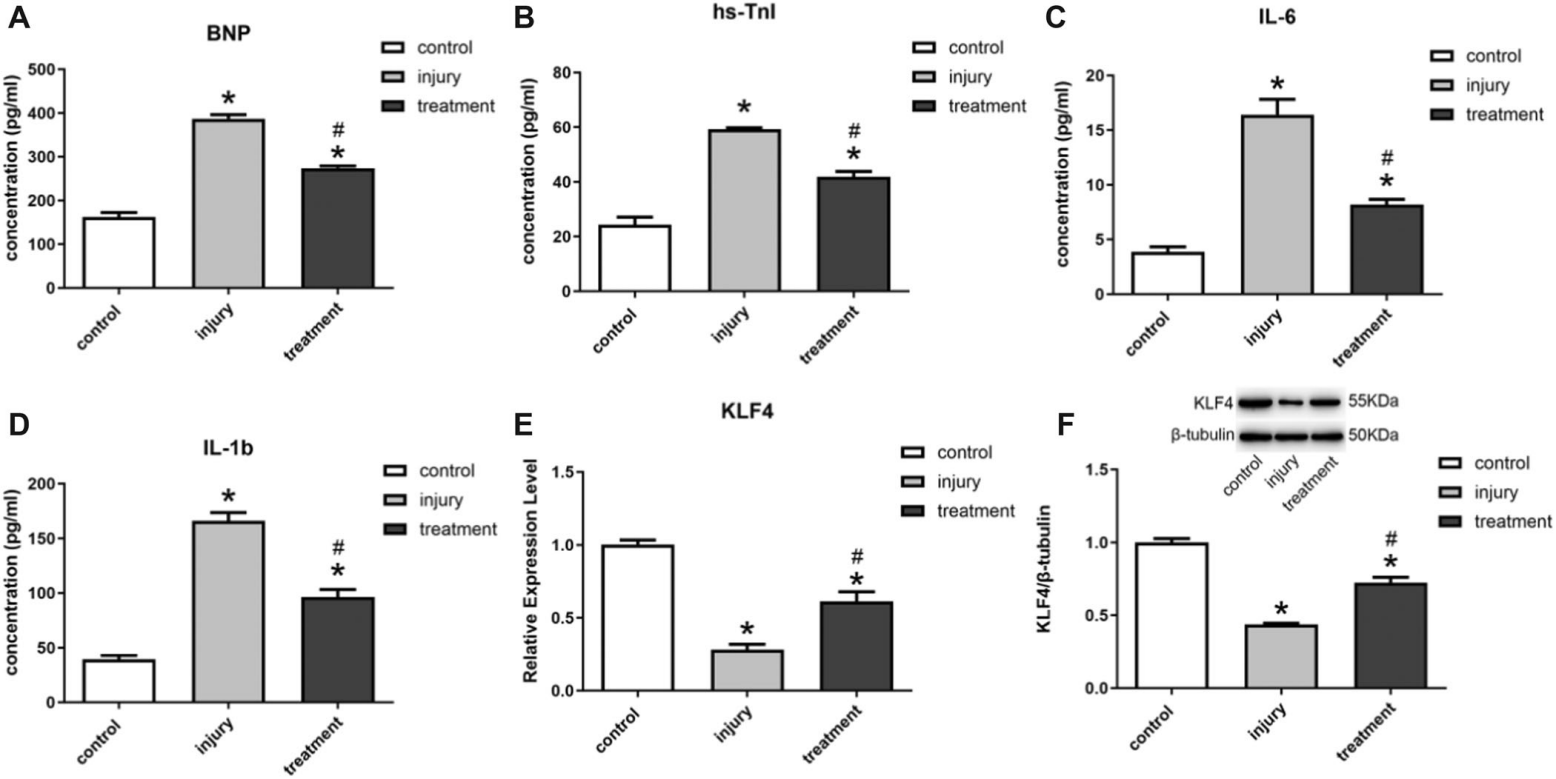
BMSC exosome treatment promoted KLF4 and suppresses inflammatory responses in HF Rats. (A‐D) ELISA for BNP, hs‐TnI, IL‐6, IL‐1b; (E) qPCR for KLF4; (F) Western blot for KLF4.

The experimental results in the SD rat model of HF demonstrated that Dox treatment induced a significant increase in BNP and hs‐TnI (Figure 5A,B), as well as increased IL‐6 and IL‐1b, in rats compared with nomal group (Figure 5C,D). The treatment of exosomes, in contrast, exhibited a significant amelioration of the impaired state, as evidenced by the decreased expression of BNP, hs‐TnI, IL‐6 and IL‐1b (Figure 5A–D). Meanwhile, the expression of KLF4 in rat myocardial tissues was examined, and the results showed that exosome treatment could significantly increase the expression of KLF4 (Figure 5E,F).

### 3.6 Interference With KLF4 Expression Inhibited the Anti‐Inflammatory Effect of Exosomes

**Figure 6:**
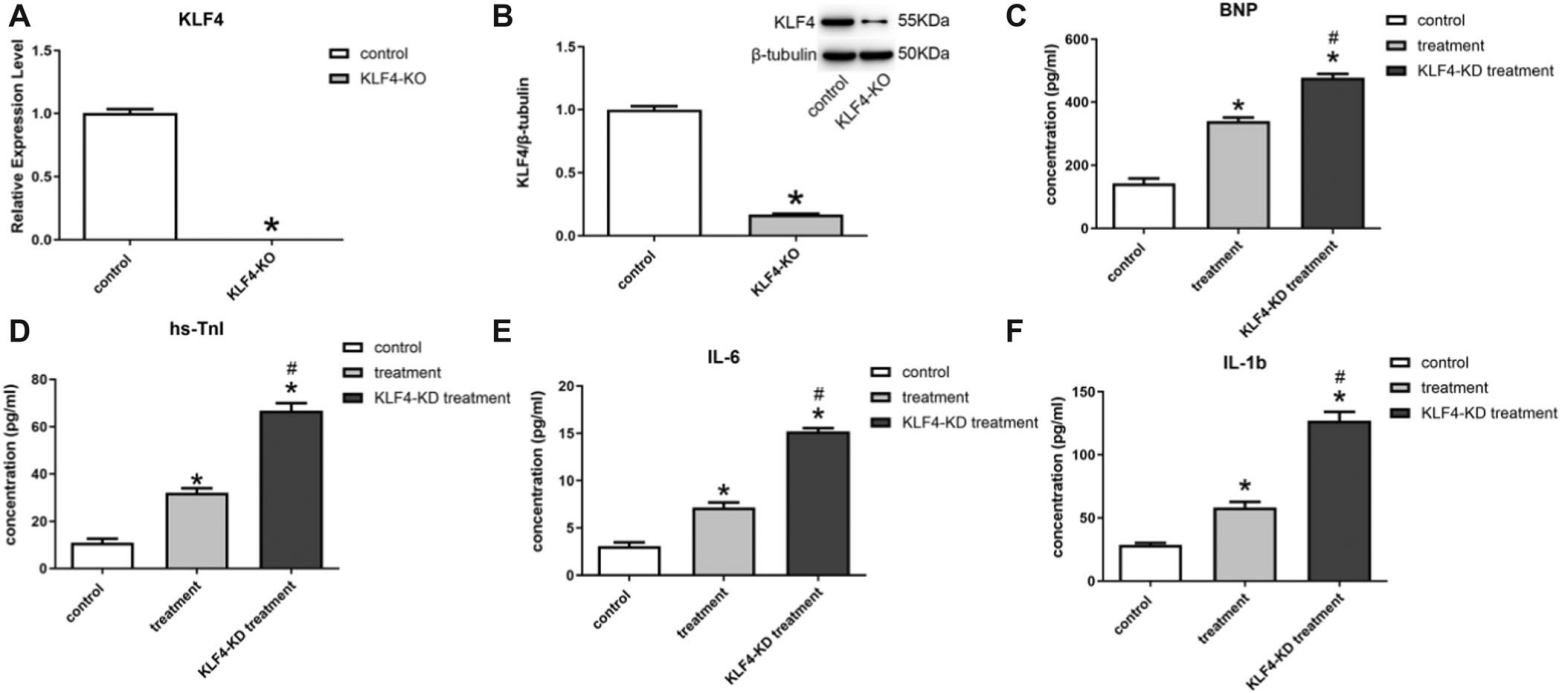
Inhibition of KLF4 reduced the therapeutic effect of exosomes in HF rats. (A) qPCR for KLF4; (B) Western blot for KLF4; (C–F) ELISA for BNP, hs‐TnI, IL‐6, IL‐1b.

This study constructed SD rats with knocked‐down KLF4 (KLF4‐KD). Experiments demonstrated that KLF4 in myocardial tissues of KLF4‐KD rats showed a substantial decrease (Figure 6A,B). Compared with normal HF rats, KLF4‐KD rats showed higher expression of indicators of myocardial damage (Figure 6C,D) and expression levels of inflammatory factors after exosomal treatment (Figure 6E,F).

## 4 Discussion

In this study, exosomes derived from BMSCs were isolated using ultracentrifugation. Through in vivo and in vitro experiments, it was observed that the exosomes exhibited a capacity to attenuate cardiomyocyte damage, reduce inflammatory cell expression, and alleviate HF injury. Additionally, the therapeutic efficacy of these exosomes may be mediated by KLF4 signaling pathway activation. Notably, downregulation of KLF4 significantly impeded their therapeutic effects.

Currently, the survival rate of HF patients in China within 5 years is still less than 50%, and the number of HF patients in the world is more than 20 million, which is still increasing at the rate of 2 million per year. More critically, current pharmacological and surgical interventions have proven ineffective in treating HF, thus highlighting the urgent need for novel therapeutic strategies and efficacious targets for HF. The therapeutic effect of MSC is primarily achieved through its paracrine action, which involves the migration of MSCs to damaged tissues and subsequent release of a plethora of cytokines, growth factors, and chemokines. These bioactive molecules collectively contribute to tissue repair and regeneration, thereby facilitating the intrinsic self‐repair mechanisms [28, 29, 30, 31]. Compared with direct cell transplantation, exosomes have stronger migration and penetration abilities, as well as lower immunogenicity [32]. MSC‐derived exosomes have been useful in the treatment of a variety of diseases, but there is a lack of sufficient in‐depth studies on the therapeutic effects and mechanism of action of HF [33]. The differentiation potential and therapeutic effects possessed by different types of MSC differ, and thus there are also differences in physical properties and therapeutic effects between exosomes of different MSC origins. To date, no comprehensive comparative analysis has been conducted on exosomes derived from various sources of MSCs, which leaves the therapeutic efficacy in different diseases uncertain. The selection of BMSC‐derived exosomes as the research target in this study was primarily based on their closer resemblance to the hematopoietic environment, enhanced cell growth and differentiation abilities, and stronger regenerative potential, which may potentially yield more pronounced effects in HF treatment. In the upcoming study, we will conduct a comprehensive comparison and analysis of the therapeutic effects of various types of MSC exosomes. Meanwhile, to ensure the quality of exosome extraction, ultracentrifugation was employed to extract exosomes from BMSCs. Subsequently, crucial physical properties such as morphology and particle size were meticulously examined, revealing their possession of a characteristic cup‐shaped structure and appropriate particle size. Furthermore, these exosomes exhibited an array of typical marker proteins associated with exosomes. In subsequent studies, we aim to further elucidate the in vivo migration patterns of exosomes and their residence time within myocardial tissues following injection, as well as conduct a comparative analysis between the effects of single and multiple injections.

In this work, Dox was utilized as an induction reagent for HF, and H9C2 cells and SD rats were selected as the in vitro and in vivo study targets, respectively. The administration of exosomes was performed via tail vein injection to minimize potential harm to the rats. Subsequently, the study utilized exosomes for the treatment of HF, and the findings revealed a significant increase in the expression of myocardial damage markers BNP and hs‐TnI, as well as inflammatory cytokines TNF‐α, IL‐6, IL‐1b, sST2, and Gal‐3 in the Dox‐induced injury group compared to the control group. These results indicate a severe impairment of cardiomyocyte′s normal physiological state due to Dox treatment. The administration of exosomes derived from BMSCs in the treatment group significantly ameliorated the impaired condition of cardiomyocytes and myocardial tissues, leading to a downregulation in the expression of markers associated with myocardial damage and inflammatory factors. The reduced expression of KLF4 significantly decreased the therapeutic effect of exosomes. The findings suggest that exosomes derived from BMSCs possess specific therapeutic functions for HF and hold promise as a novel therapeutic approach for HF. Additionally, the KLF4 may play a crucial role in regulating these functions as a key protein. However, in comparison to previous studies, the current experiment not only confirms the superior therapeutic effect of exosomes on HF, but also provides further evidence that this physiological effect is primarily achieved through the reduction of inflammatory response in myocardial tissues. Additionally, it suggests that the KLF4 factor may serve as a crucial protein in the therapeutic pathway, thereby offering valuable insights for future HF treatment using exosomes.

It is important to note that the induction of HF in rats by Dox does not accurately represent all forms of HF diseases, which imposes certain limitations on this experiment for studying novel modalities and targets for HF treatment. Therefore, future studies will also explore the application of this therapeutic modality to other models of cardiovascular diseases to determine its universality. The transcription factor KLF4 plays a crucial role in various cellular processes and the pathogenesis of diseases [34, 35]. This study reveals a significant decrease in the expression level of KLF4 among HF patients compared to the normal population, indicating its pivotal involvement in HF development and progression. Consequently, KLF4 emerges as a promising therapeutic target for HF. Subsequently, through in vitro and in vivo experiments, we observed a significant reduction in the therapeutic efficacy of exosomes on HF when KLF4 expression was knocked down in cardiomyocytes. This finding provides evidence for the involvement of KLF4 in the physiological process of exosomal treatment for HF and highlights its crucial regulatory role. Although the specific mechanism by which exosome treatment activates high expression of KLF4 in cardiomyocytes remains unclear, further research findings from subsequent experiments are required. However, based on the results of our present experiments, we propose a hypothesis that KLF4 may play a role in regulating inflammatory responses in cardiomyocytes and alleviating heart failure by downregulating the expression levels of inflammatory factors.

## 5 Conclusion

In this study, exosomes derived from BMSCs were utilized as therapeutic agents for Dox‐induced H9C2 cells and SD rats, in conjunction with molecular biology techniques. The present study demonstrated that exosomes derived from BMSCs were able to exert a significant effect on alleviating HF and reducing inflammation levels by increasing KLF4 in cardiomyocytes, providing a new direction and target point for the treatment of HF. However, the specific mechanisms through which exosomes regulate KLF4 in cardiomyocytes, as well as the role of KLF4 in orchestrating the inflammatory response and damage repair in these cells, remain inadequately understood, highlighting the need for further research.

## Author Contributions

**Yutong Han:** conceptualization, project administration, resources, writing – original draft, writing – review and editing. **Yang Bi:** data curation, resources, writing – original draft, writing – review and editing. **Donghai Zhang:** methodology, resources, writing – original draft, writing – review and editing. **Yubao Liu:** funding acquisition, project administration, writing – original draft, writing – review and editing.

## Ethics Statement

The study was approved by the Ethics Committee of the Second Affiliated Hospital of Qiqihar Medical University (No. 202402003‐01).

## Consent

The authors have nothing to report.

## Conflicts of Interest

The authors declare no conflicts of interest.

## Data Availability

The data that support the findings of this study are available from the corresponding author upon reasonable request.
